# The international student-athlete life: risk factors, stressors, and mental health; a brief research report

**DOI:** 10.3389/fspor.2026.1712505

**Published:** 2026-04-07

**Authors:** Dimitra Galbierz, Anastasios Kaburakis, Jesse J. Helton

**Affiliations:** 1 School of Social Work, Saint Louis University, Saint Louis, MO, United States; 2 Department of Management, Chaifetz School of Business, Saint Louis University, Saint Louis, MO, United States

**Keywords:** International student-athletes, mental health, depression, anxiety, risk factors

## Abstract

**Introduction:**

International Student-Athletes (ISAs) represent a unique population in collegiate sports, facing distinct challenges that may impact their mental health beyond those experienced by domestic athletes. While ISAs contribute significantly to NCAA programs, limited quantitative research exists on their specific risk factors, stressors, and mental health needs. This study examined factors affecting ISAs’ wellbeing and their utilization of mental health resources.

**Methods:**

A cross-sectional survey was distributed to ISAs at Division I and II universities through athletic directors and coaches. The anonymous online questionnaire assessed demographics, depressive symptoms (PHQ-2), anxiety symptoms (GAD-2), relationship satisfaction with coaches/teammates/staff, life domain satisfaction, homesickness, discrimination experiences, and mental health service utilization. Chi-square analyses examined associations between variables and mental health symptoms.

**Results:**

Among 207 participants (87% female and 83% European origin), 18% reported depressive symptoms and 25% reported anxiety in the previous 2 weeks. Mental health service utilization was 41%. Significant associations emerged between mental health symptoms and dissatisfaction with coaches, teammates, staff, diet, sleep, and social life. Homesickness affected 63% and was linked to higher depression and anxiety rates. Twenty percent experienced discrimination and 27% reported food insecurity.

**Discussion:**

ISAs face substantial mental health challenges, with relationship quality emerging as a critical factor. The findings highlight the importance of supportive coach-athlete and teammate relationships for ISA wellbeing. Future research should explore cultural and gender variations while institutions should consider specialized mental health support and culturally informed training for athletic staff working with this population.

## 1 Introduction

Over the past few decades, there has been a gradual increase of research focusing on International Student Athletes (ISAs), who come to the United States from all areas across the globe. Latest reports show a rapid decrease in international student enrollment, which is projected to significantly affect U.S. Higher Education and the job market (1). Yet nearly 25,000 ISAs receiving athletic scholarships and competing in NCAA Divisions I and II from all around the world (2) underscore ISAs’ critical impact on the U.S. collegiate sports ecosystem, thus rendering them irreplaceable. Furthermore, it is not uncommon for college teams to recruit the best talent to win, which ultimately could give them access to various resources. With highly talented, differentiating ISAs, college sports programs can compete for conference, tournament, and national championship titles, payouts, creating sponsorship and donor opportunities, strengthening the team's, athletic department's, and entire institution's brand image. Recruiting ISAs to enhance college teams’ chances to win has been both controversial and a critically important strategy toward gaining a competitive advantage (3–6, 49).

This research was conducted over the past year, when there were increased numbers of ISAs across most intercollegiate sports in the US (2). Over the past decade, but more specifically since COVID-19, several studies have attempted to study mental health and college athletes (7–9, 47). While no comprehensive study focused on ISAs mental health heretofore, important findings from previous research on mental health and college athletes informed this research project. Prior contributions ranged from college athletes seeking help and support systems (10–17, 45, 48) to the contemporary and complex issues of the transfer portal and NIL further impacting ISAs’ mental health (18–20). Studies also encompassed literacy and understanding of mental health conditions (21), balancing demands of competitive sport, academics, and mental health (22, 23), addressing cohesion and teammates’ relationships (24, 42), occasionally addressing modulating factors, such as race and ethnicity (25, 50), as well as gender (26, 27). All these factors and the additional ones ISAs need to manage as highly regulated (and restricted) “legal aliens or alien students” (28, 44), present particular obstacles and risk factors. It also offersopportunities for research into ways to transition from mental health problems such as depression and anxiety to treatment solutions and resources, optimally available and particularly attending to ISAs needs, in scholarships-awarding NCAA institutions.

Although research indicates that collegiate student-athletes in the U.S. constitute a unique population at risk for mental health challenges due to the complex demands associated with balancing athletic commitments and academic responsibilities (15, 29–31), academia and practice have yet to fully embrace the need for interventions and support tailored specifically to the experiences of ISAs. ISAs face barriers to their wellbeing not experienced by domestic athletes, such as language barriers, acculturation stress, homesickness, and even legal stressors due to navigating procedurally entangled visa and taxation issues (32–35, 46). Food insecurity as previously defined by inadequate access to nutritionally adequate food, while acquired in socially acceptable ways to support an active and healthy life (51), has been shown to be an issue for collegiate student-athletes (36, 43, 52, 53), but overall findings of prevalence and how ISAs are impacted remain understudied (37). In addition, ISAs are currently preempted from fully benefitting from recent Name, Image, and Likeness (NIL), and revenue share opportunities due to work restrictions on student visas placed by the Department of Homeland Security (38). In a recent qualitative meta-synthesis (54), the authors attempted to synthesize the existing literature related to the adjustment and acculturation issues ISAs experience, and to explore the research gaps that generally exist while advocating for this population.

The existing literature still lacks empirical evidence on what makes the ISA experience unique, whether these athletes are satisfied with their life and routine on and off-campus, and what specific risk factors may impact their overall mental health. The current study's aim is to further explore the ISA experience in U.S. college athletics and contribute to the existing literature, as well as identify barriers to seeking support and coping with mental health-related issues.

## 2 Method

Data was collected using a cross-sectional design and the survey was distributed via email to almost 7,000 administrators and coaches at NCAA Division I and Division II institutions for further dissemination to each institution's ISAs, to examine ISAs mental health. The athletes were invited to complete an anonymous online survey exploring stressors, coping mechanisms, institutional support systems, and that effort yielded 207 completed responses from ISAs. We are unaware of how many surveys were forwarded to ISA and cannot calculate a response rate. Participation was voluntary, confidential, and could be withdrawn at any time without penalty. The study was approved by the authors’ Institutional Review Board (IRB# 33984).

ISA demographics, including age, gender, race, country of origin, class, and type of sport they played were measured. Depressive and anxiety symptoms were assessed using the Patient Health Questionnaire (PHQ-2) and the Generalized Anxiety Disorder (GAD-2) scales. Both the PHQ-2 (39) and the GAD-2 (40) show strong reliability, with Cronbach alphas ranging from .71 to .85. The scales asked how often the athlete felt bothered by the following feelings in the past 2 weeks: (1) having little interest, (2) down or depressed, (3) anxious or nervous, and (4) not being able to stop worrying. Respondents answered on a 4-point scale. If an athlete responded by being bothered by one of the four feelings “nearly everyday” or “more than half the days”, they were coded as having a mental health symptom. Respondents were also asked if they felt homesick, as well as asked to rate their satisfaction with their coaches, teammates, team staff, their social life, diet, finances, and sleeping habits. Furthermore, the respondents were asked if they have ever been discriminated against due to their cultural background, ethnicity, sex, gender, race, or religious beliefs and if they have ever experienced food insecurity. Finally, participants were asked if they had ever utilized any mental health services and their level of satisfaction with that service. Differences were assessed using Pearson chi-square analyses.

## 3 Results

Table 1 presents descriptive statistics for the sample (*N* = 207). Overall, 18% of athletes reported depressive symptoms, 25% reported anxiety, and 31% endorsed either depression or anxiety. Fourteen percent reported having been assessed for a mental health disorder, and 41% indicated utilizing mental health services. Satisfaction with relationships was generally high, with 57% of athletes reporting satisfaction with coaches, 67% with teammates, and 76% with trainers or medical staff. Satisfaction with life domains was somewhat lower: 38% reported satisfaction with finances, 28% with diet, 48% with sleep, and 49% with their social life outside of sports. Homesickness was common, with 34% somewhat homesick and 29% homesick. Regarding social and economic barriers, 27% of athletes reported food insecurity, and 20% reported experiencing discrimination due to cultural or ethnic background.

**Table 1:** Descriptive statistics of the ISA sample.

Variables	%	*n*	*M*	SD
Mental health variables				
Depression	18	38		
Anxiety	25	52		
Depression or anxiety	31	65		
Assessed for mental health disorders	14	28		
Utilized mental health services	41	75		
Relationship satisfaction				
Coaches				
Not satisfied	15	30		
Somewhat satisfied	29	59		
Satisfied	57	117		
Teammates				
Not satisfied	9	19		
Somewhat satisfied	24	50		
Satisfied	67	138		
Trainers/Medical staff				
Not satisfied	6	13		
Somewhat satisfied	18	37		
Satisfied	76	157		
Life satisfaction				
Finances				
Not satisfied	21	43		
Somewhat satisfied	41	85		
Satisfied	38	79		
Diet				
Not satisfied	31	65		
Somewhat satisfied	41	84		
Satisfied	28	58		
Sleep habits				
Not satisfied	19	39		
Somewhat satisfied	33	68		
Satisfied	48	100		
Social life outside sports				
Not satisfied	18	38		
Somewhat satisfied	32	67		
Satisfied	49	102		
Homesick				
Not homesick	37	77		
Somewhat homesick	34	71		
Homesick	29	59		
Social and economic barriers				
Food insecure	27	56		
Discriminated against	20	42		
Demographic variables				
Age			20.4	1.8
Class				
Freshman	27	56		
Sophomore	21	44		
Junior	20	41		
Senior	18	38		
Graduate/Alumni	14	28		
Gender (female)	87	181		
Race/Ethnicity (non-white)	15	32		
Geographic origin (European)	83	172		
Sport				
Volleyball	51	106		
Tennis	7	15		
Basketball	14	28		
Track	5	10		
Swimming and diving	6	13		
Other	17	35		

*N* = 207. Percentages and frequencies are reported for categorical variables; means and standard deviations are reported for continuous variables. Mental health variables are coded as 1 = present, 0 = absent. European origin is coded as 1 = Europe, 0 = all other countries.

The sample was predominantly female (87%), with a mean age of 20.4 years (SD = 1.8). Class standing was distributed across undergraduates (freshman 27%, sophomore 21%, junior 20%, senior 18%) and graduate/alumni (14%). Most athletes were of European origin (83%), and 15% identified as non-White. The largest sport represented was volleyball (51%), followed by basketball (14%), other sports (17%), tennis (7%), swimming/diving (6%), and track (5%). Table 2 presents the mental health outcomes of international student-athletes (ISAs) according to their relationship status, life satisfaction, perceived barriers, and demographic characteristics.

**Table 2:** Mental health by relationship and life satisfaction, barriers, and demographics of the ISAs.

Variables	Depression	Anxiety
Relationship satisfaction		
Coaches		
Not satisfied	40**	33*
Somewhat satisfied	24	36
Satisfied	10	17
Teammates		
Not satisfied	32**	47*
Somewhat satisfied	30	32
Satisfied	12	20
Trainers/Medical staff		
Not satisfied	62**	38
Somewhat satisfied	14	27
Satisfied	16	24
Life satisfaction		
Finances		
Not satisfied	28	35
Somewhat satisfied	13	22
Satisfied	19	23
Diet		
Not satisfied	26*	37*
Somewhat satisfied	19	23
Satisfied	9	16
Sleep habits		
Not satisfied	36**	31
Somewhat satisfied	19	32
Satisfied	11	18
Social life outside sports		
Not satisfied	34**	39*
Somewhat satisfied	24	30
Satisfied	9	17
Homesick		
Not homesick	8**	17**
Somewhat homesick	24	21
Homesick	25	41
Social and economic barriers		
Food insecure		
Yes	23	32
No	16	23
Discriminated against		
Yes	24	38*
No	17	22
Demographic variables		
Class		
Freshman	23	25
Sophomore	16	25
Junior	12	20
Senior	21	29
Graduate/Alumni	18	29
Gender		
Female	19	28**
Male	11	4
Race/Ethnicity		
Non-White	16	25
White	19	25
Geographic origin		
Europe	19	25
Other	17	26
Sport		
Volleyball	19	29
Tennis	27	20
Basketball	14	21
Track	20	20
Swimming and Diving	23	31
Other	14	17

*p < .05.** *p* < .01.

Chi-square analyses revealed significant associations between satisfaction with coaches and depressive symptoms, *χ*^2^(2, *N* = 206) = 15.58, *p* < .001. Forty percent of athletes who were not satisfied with their coaches reported depression, compared to 24% of those somewhat satisfied and 10% of those satisfied. Similarly, anxiety symptoms varied significantly by coach satisfaction, *χ*^2^(2, *N* = 206) = 8.59, *p* = .014, with the highest prevalence among athletes who were less satisfied. Satisfaction with teammates was also associated with depression, *χ*^2^(2, *N* = 207) = 10.10, *p* = .006. Thirty-two percent of athletes who were not satisfied with teammates reported depression compared to 30% of those somewhat satisfied and 12% of those satisfied. Anxiety showed a similar pattern, *χ*^2^(2, *N* = 207) = 8.52, *p* = .014, with 47% of those not satisfied reporting anxiety, compared to 32% and 20% among somewhat satisfied and satisfied athletes, respectively. Satisfaction with trainers and medical staff was strongly associated with depression, *χ*^2^(2, *N* = 207) = 17.37, *p* < .001. More than 60% of athletes who were not satisfied with trainers or medical staff reported depressive symptoms, compared to 14% of those somewhat satisfied and 16% of those satisfied.

Satisfaction with diet was significantly related to depression, *χ*^2^(2, *N* = 207) = 6.33, *p* = .042. Among those not satisfied with their diet, 26% reported depressive symptoms compared to 19% of those somewhat satisfied and 9% of those satisfied. Diet satisfaction was also associated with anxiety, *χ*^2^(2, *N* = 207) = 7.94, *p* = .019; 37% of athletes who were not satisfied with their diet reported anxiety compared to 23% of those somewhat satisfied and 16% of those satisfied. Sleep satisfaction was also significantly associated with depression, *χ*^2^(2, *N* = 207) = 11.64, *p* = .003. More than one-third (36%) of athletes who were not satisfied with their sleep reported depressive symptoms, compared to 19% of those somewhat satisfied and 11% of those satisfied. Satisfaction with social life outside of sports was significantly associated with depression, *χ*^2^(2, *N* = 207) = 13.92, *p* = .001, and anxiety, *χ*^2^(2, *N* = 207) = 8.83, *p* = .012. Over one-third (34%) of those not satisfied with their social life reported depression and 39% reported anxiety, compared to 9% and 17% of those satisfied, respectively. Homesickness was significantly associated with both depression, *χ*^2^(2, *N* = 207) = 9.18, *p* = .010, and anxiety, *χ*^2^(2, *N* = 207) = 10.97, *p* = .004. Athletes who reported being somewhat or very homesick endorsed substantially higher rates of depression (24%–25%) and anxiety (21%–41%) compared to those who reported not being homesick (8% depression; 17% anxiety).

Discrimination experiences were associated with higher anxiety, *χ*^2^(1, *N* = 207) = 4.72, *p* = .030. Nearly 40% of athletes reporting discrimination endorsed anxiety, compared to 22% of those who did not report discrimination. Gender was also significantly associated with anxiety, *χ*^2^(1, *N* = 207) = 7.15, *p* = .007. Female athletes reported substantially higher rates of anxiety (28%) than male athletes (4%). No significant associations were observed between mental health symptoms and class year, race/ethnicity, geographic origin, or sport.

## 4 Discussion

Our results revealed that nearly one-third of ISAs experienced depressive or anxiety symptoms, with particularly strong associations between mental health challenges and dissatisfaction in key relationships with coaches, teammates, and athletic support staff. The findings underscore that relationship quality emerged as a more critical predictor of mental health than other factors outside athletic departments, suggesting that ISAs prioritize interpersonal connections within their athletic environment [aligned with (24) on cohesion with teammates] over other aspects of their college experience. These findings regarding ISAs’ dissatisfaction with coaches and athletic staff echo what Charitonidi and Kaburakis (32) describe as a lack of guidance and support from coaching staff, reinforcing that relationship quality is a critical predictor of mental health outcomes for this population. Our findings compliment those of Hayashi and colleagues (41), which called for more research attempting to examine ISAs particular challenges, need for mental health support, more resources, and more attention to this demographic's well-being. Especially at a time when retaining top-tier talent may be crucial for sustained success in high-stakes NCAA D1 athletic competition, additional research and data from ISAs may prove instrumental in enhancing their intercollegiate athletics experience.

Even though this study provides significant insight into the life of ISAs and the factors that contribute to their mental health and well-being, there certainly were several limitations to note: The sample was limited (*N* = 207), predominantly female (87%) and largely composed of athletes from Europe (83%), specifically playing volleyball (51%), which reflects the inherent challenges of recruiting ISAs for mental health research, while recruitment efforts may have been constrained by institutional gatekeeping to pass the survey along to ISAs, time demands associated with athletics and academics, and potential stigma surrounding mental health disclosure. The gender, sport, and ethnicity of the athletes limit the generalizability of the findings to the broader ISA population, especially male, non-European, sports heavily recruiting ISAs, as well as those in potentially underrepresented sports, both team sports (e.g., ice hockey, soccer, water polo, etc.) and individual sports (e.g., rowing, tennis, track and field, swimming and diving, etc.). Furthermore, the study's cross-sectional design only captures experiences at a single point in time, does not allow for conclusions about changes in mental health over time, and its nature prevents the authors from establishing any causal relationships in this study. All data were collected through a self-report survey, which is subject to response bias, especially regarding sensitive topics like mental health, and those experiencing higher levels of distress may have been either more motivated to participate due to personal relevance or less likely to engage due to avoidance or discomfort. Cultural norms surrounding mental health could have also affected the accuracy or the extent responses could be compared across participants from different regions. Additionally, even though the study used valid screening tools for depressive and anxiety symptoms, these likely do not capture the full spectrum of mental health and overall well-being in ISAs. Finally, recruitment also relied heavily on the athletic directors’ and coaches’ willingness to share the study with their athletes, and the athletes’ willingness to complete the survey anonymously. Nevertheless, the study's results not only confirm previous concerns about ISAs’ mental health risks, but also reveal new information regarding the underlying social, environmental, and relational stressors impacting this population.

Among others, strengths of this research include the sample size in this study, which appears to be the largest quantitatively of an independent non-NCAA-sponsored academic group. Albeit still at an earlier stage of what the authors aspire to be the most comprehensive quantitative and qualitative research project surveying ISAs, the fact this study yields more than 200 ISAs responses in a voluntary and not incentivized study is significant in itself. Moreover, the fact that approximately one fifth of the population in this study reported depressive symptoms, and one fifth of respondents also established feelings of being discriminated against, would need to be underscored and further investigated in ensuing research streams. One fourth reporting anxiety is also significant, especially as anxiety is directly associated with poor athletic and academic performance. One surprising finding was that more than 40% of respondents self-reported utilizing mental health resources, which is both promising regarding the positive impact NCAA programs can have on ISAs, as well as somewhat alarming, in respect to the volume of ISAs identifying a need to use such support services. On the positive side, relations with coaches, teammates, and athletic training/medical staff ranged from satisfactory to highly positive; this outcome was especially significant considering the importance ISAs emphasized in regard to these interrelationships and their impact on ISAs mental health. Life domains’ responses certainly merit further examination, particularly pertaining to food insecurity and diet concerns. Support staff, especially trainers and nutrition/dietetics professionals need to be involved in such research and align their work with the administrative and coaching staff, to better address such reported ISAs’ needs. On the latter, arguably the most significant finding of this study has been the importance of ISAs’ relationships with coaches, teammates, and support staff. Dissatisfaction with these key actors in ISAs’ experiences was related to mental health issues at higher rates than life domains. Stated otherwise, ISAs prioritized their relationships with their coaches and teammates, over any other part of their college experience. To date, this research stream is the seminal study of ISAs and mental health, a significant population for NCAA institutions’ recruitment and differentiating competitive impact. The authors of this study hope to inspire further works in the sensitive space of mental health research, particularly impacting international students and athletes, given recent geopolitical developments affecting their access to (and experiences in) US Higher Education.

## 5 Conclusion

Although preliminary, our findings indicate that ISAs face significant challenges related to depressive and anxiety symptoms. The results underscore the critical importance of relationship-building. Given that ISAs relocate from their home countries to pursue both academic and athletic careers in the U.S., and spend the majority of their time with teammates, coaches, and athletic staff, their satisfaction with these relationships and environments is vital to their well-being.

Future research should aim to better understand the risk factors contributing to ISAs’ mental health challenges, with particular attention to variations across ethnicity, cultural background, gender, and sport. Such insights can help build a more nuanced understanding of the ISA experience. Moreover, institutions should consider hiring mental health professionals, who are specifically trained to work with international populations, to more effectively support these students. Athletic department staff and coaches also play a pivotal role in the adjustment and overall experience of ISAs, and should therefore be proactive in cultivating culturally informed and supportive relationships—starting as early as the recruitment process.

## Data Availability

The datasets presented in this article are not readily available because this is an ongoing data study collection. Requests to access the datasets should be directed to Dimitra Galbierz, dimitra.galbierz@health.slu.edu.
